# Performance of GPS units for deployment on semiaquatic animals

**DOI:** 10.1371/journal.pone.0207938

**Published:** 2018-12-06

**Authors:** Lia Schlippe Justicia, Frank Rosell, Martin Mayer

**Affiliations:** 1 Faculty of Technology, Natural Sciences, and Maritime Sciences, Department of Natural Sciences and Environmental Health, University of South-Eastern Norway, Bø i Telemark, Norway; 2 Faculty of Biology, University of Barcelona, Barcelona, Spain; 3 Department of Bioscience, Aarhus University, Aarhus, Denmark; Fred Hutchinson Cancer Research Center, UNITED STATES

## Abstract

Global Positioning System (GPS) technology is widely used in wildlife research to study animal movement and habitat use. In order to evaluate the quality and reliability of GPS data, the factors influencing the performance of these devices must be known, especially for semiaquatic species, because terrestrial and aquatic habitat might affect GPS performance differently. We evaluated the location error and fix success rate of three GPS receiver models in stationary tests and on a semi-aquatic mammal, the Eurasian beaver (*Castor fiber*). The location error during stationary tests was on average 15.7 m, and increased with increasing canopy closure, slope, and horizontal dilution of precision, potentially leading to the erroneous classification of GPS positions when studying habitat use in animals. In addition, the position of the GPS antenna (flat versus 90° tilted) affected the location error, suggesting that animal behavior affects GPS performance. The fix success rate was significantly higher during stationary tests compared to when GPS units were deployed on beavers (94% versus 86%). Further, GPS receivers did not obtain any positions underwater and underground, the latter potentially allowing the estimation of activity periods in animals that use lodges or burrows as shelter. We discuss the possibilities for data screening, the use of buffer zones along the shoreline, and combination with other data loggers to avoid the erroneous classification of GPS positions when studying habitat use.

## Introduction

Global positioning system (GPS) technology allows the remote data collection of animal positions and movements [1, 2]. Therefore, GPS units are a valuable tool for estimating animal home range sizes [3, 4], studying habitat use and resource selection [5, 6], as well as movement patterns and migratory routes [7–9]. The use of GPS telemetry has several technical advantages compared to conventional very high frequency (VHF) triangulation techniques [10] or Argos satellite positioning [11], as it is more accurate, and available for 24h a day with position updates available in rapid succession [12]. Since its initial development, GPS tracking has significantly improved due to the reduction in size and weight, increased battery life, lowered costs, and large capacity of data storage [13]. These improvements now allow us to track small species such as hedgehogs (*Erinaceus europaeus*) [14] and ovenbirds (*Seiurus aurocapilla*) [15].

However, raw data obtained from these methods can contain error and bias that require rigorous and objective testing [16]. To estimate a location, a GPS device must receive information from ≥3 satellites, and this reliance on satellites can cause two types of errors in the GPS performance. The first one is the location error (LE), defined as the Euclidian distance between each GPS-generated location and the true location, which occurs when the GPS unit records a location that is inaccurate [17]. The second error type is unsuccessful fix acquisition that occurs when the GPS cannot acquire signals from enough satellites to generate a location estimate, which results in missing location data [17, 18]. This is measured as fix success rate (FSR), defined as the number of successful fix attempts divided by the total attempted fixes. The magnitude of both errors depend on technological [19–21], environmental [18, 22–24] and behavioral factors [16, 25]. The main technological factors affecting LE and FSR are the number of satellites and satellite geometry, expressed as dilution of precision (DOP) including horizontal DOP (HDOP). Smaller DOP values indicate wider spacing between satellites, which potentially minimizes triangulation error and, therefore, increases position accuracy (i.e., lower LE) [18]. Lewis, Rachlow [26] suggested removing GPS positions with DOP values > 5 and 2-D positions (3 satellites) to increase accuracy.

Several studies have assessed the effects of different habitat and climate variables on GPS fix acquisition, such as habitat type, topography, water submersion, precipitation, cloud cover, and vegetation characteristics (tree height, tree diameter and canopy closure) [22–24, 27]. For example, studies on white-tailed deer (*Odocoileus virginianus*) and wolves (*Canis lupus*) found that vegetation closure had a negative effect on GPS accuracy [28], because it can block or reflect satellite signals, leading to higher LE values [29, 30]. Similar effects can occur in rugged terrain and built-up areas, leading to a reduced FSR [31]. Further, water submersion was shown to negatively affect GPS accuracy due to the inability of GPS signals to properly propagate in water [32]. For example, Costa, Robinson [33] found that the LE was smaller in sea lions (*Zalophus californianus; Z*. *wollebaeki)* and fur seals (*Arctocephalus pusillus pusillus; A*. *p*. *doriferus)*, which spend more time on the surface and make shorter dives compared to species that make long dives followed by shorter surface intervals like elephant seals (*Mirounga angustirostris*). Moreover, the behavior of tracked animals can bias the FSR and LE by causing variations in signal reception due to body obstruction and changes in antenna position [27]. In grizzly bears (*Ursus arctos*), GPS fix success decreased when individuals were bedding, thereby moving the GPS antenna toward the side or ground, leading to a reduced reception of satellite signals [34]. The error introduced by animal behavior is still largely unexplored because of the difficulties of setting up suitable field tests [35]. Conversely, animal behavior itself can help to identify erroneous locations. For example, Bjørneraas, Van Moorter [36] developed a method for screening GPS data based on unrealistic species-specific speed and distance travelled between consecutive locations.

In this study, we evaluated factors affecting FSR and LE of three models of GPS receivers along riparian habitat. Riparian habitat is important for many semiaquatic species, including insects [37], amphibians [38], reptiles [39], birds [40] and mammals [41, 42], and acts as migration route and corridor connecting habitat patches [43]. A specific challenge when studying habitat use in semiaquatic animals is the assignment of an individual being in water or on land, respectively, requiring a high GPS accuracy. However, to our knowledge, the performance of GPS units in riparian habitat was only investigated in one study using Eurasian otters (*Lutra lutra*) [32].

We hypothesized that 1) habitat variables (canopy closure, slope, water submersion and being underground), 2) technical variables (HDOP and the number of satellites), and 3) antenna angle (as crude measure of animal body posture) would affect LE and FSR. We predicted that GPS performance would decrease (i.e., increased LE and decreased FSR) with 1) increasing canopy closure, 2) slope, and 3) when GPS units were underwater and underground due to GPS signal obstruction. Further, we predicted GPS performance to increase with 4) increasing number of satellites and lower HDOP values, and 5) when the antenna faced toward the sky compared to a tilted antenna position. In addition, we tested 6) if FSR depended on weather conditions, i.e., temperature and precipitation. Finally, we compared the FSR obtained from stationary tests to the FSR calculated from GPS data obtained from a semiaquatic mammal, the Eurasian beaver (*Castor fiber*) to investigate the influence of animal behavior.

## Materials and methods

### Study area and species

The study area was located in Telemark county, southeast Norway (59°23’ N, 09°09’ E) and consisted of two connected rivers, the Gvarv and Saua, which both empty into Lake Norsjø. The climate in the area is cool continental with a mean annual temperature of 4.6 °C and an average annual rainfall of 790 mm [44]. The area is dominated by semi-agricultural land, and riverbanks are lined with riparian woodland structures [45].

We previously deployed GPS units on Eurasian beavers (hereafter, beaver) to study habitat selection and spatial movement patterns [46–48]. Beavers are large, nocturnal, herbivorous rodents [49] that inhabit freshwater systems [50]. They move relatively close to the shoreline, both when being on land and in water, respectively [47, 51]. Further, they spend approximately half of their activity time on land and the other half in water, whereas the proportion of time spent on land and in water, respectively, varies with individual age [47]. Beavers build lodges or dens that are used as shelter during the day, although they may also return to them during nighttime [52].

### Data collection

**Fig 1:**
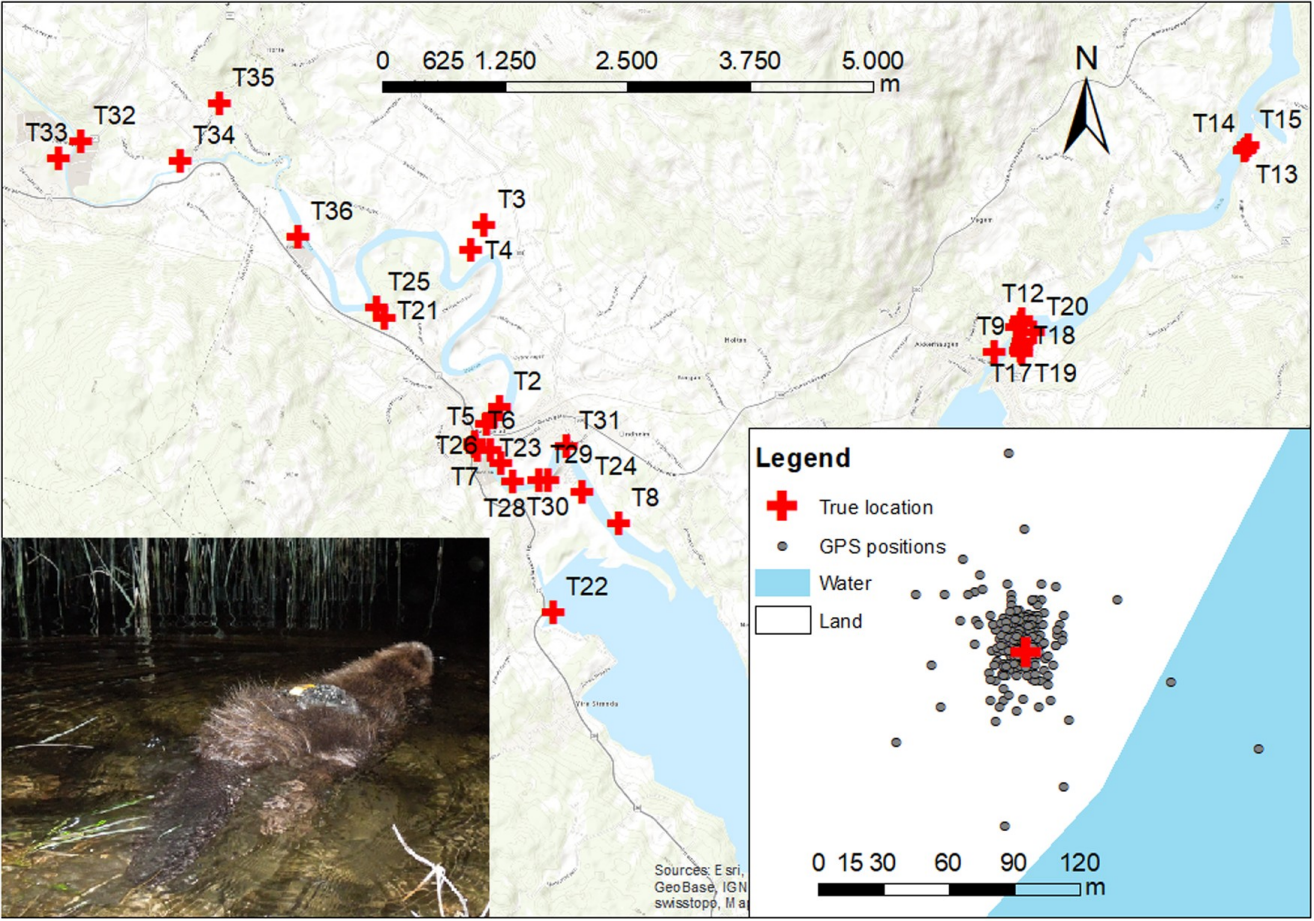
Study area in South-Eastern Norway, showing the 36 test sites (red crosses) where we tested the performance of GPS receivers (large map). The small map shows GPS locations (grey dots) for one exemplary test site, and the picture shows a Eurasian beaver (*Castor fiber*), equipped with a GPS unit on its lower back.

We conducted stationary tests from March–May 2017 and March–April 2018 with six GPS receivers of three different models (two units per model): 1) GIG 134A micro GPS, 2) PinPoint 75 micro GPS (both Sirtrack, Havelock North, New Zealand), and 3) TGB-317/315GX (Telenax, Playa del Carmen, Mexico). Models GIG 134A micro (not produced any longer) and TGB-317/315GX attempted to take a fix for 180 sec and obtained conventional GPS positions (cold start). Model PinPoint 75 micro attempted to take a fix for 70 sec, because it only stored satellite information (GPS positions were calculated post-processing). All receiver models stored data onboard and had to be recovered for data download. We selected 36 sites (Fig 1) of varying canopy closure (range: 0–95%; mean ± SD: 47 ± 34%), and slope (range: 0–56°; 17 ± 17°) to cover the variation of the habitat within our study area. Site selection considered different slope levels within three categories of vegetation height (low: 0–1.5 m, medium: 1.5–10 m, and high: >10 m), allowing us to disentangle between slope and vegetation height/canopy closure (31 different sites). Additionally, we selected five extra sites to test the GPS receivers on the water surface (two sites), underwater (two sites: 14 and 37 cm underwater, respectively) and one site underground (60 cm) in an inactive beaver den. We measured the slope, vegetation height, and canopy closure at the true GPS location for each test site. Canopy closure was measured as the proportion of sky obscured by vegetation [53], and was estimated by averaging spherical densitometer readings recorded in the four cardinal directions [54]. Further, we measured the average tree diameter measured at breast height and the basal area, defined as the amount of area occupied by tree stems, within a radius of 10 m from the true GPS location at each test site. The true location of each site was determined using a high-precision GPS unit with <1 m precision (Topcon FC-250, Topcon Positioning Systems, CA, United States, https://www.topconpositioing.com). All six GPS receivers were placed simultaneously on a 30 cm high burlap bag filled with straw, simulating a beaver, with the GPS antenna directly facing skyward (0°). We programmed the units to record one GPS position every 15 minutes between 1900 and 0700 h (49 possible fixes per night), i.e., during the activity time of beavers [52]. GPS units were programmed to not record positions during the day, because beavers are not active then [52]. To evaluate the effect of GPS antenna position (simulating animal behavior) on FSR and LE, we conducted an additional test at 11 of the 36 test sites (March–April 2018; T26-T36, S1 Table). This test was only conducted for the four Sirtrack models, because the two Telenax units were lost during fieldwork the previous year. We placed the GPS receivers at each site for two consecutive days, one day positioned at 0° (GPS antenna facing directly skyward), representing swimming/being in water, and one day at 90°, representing sitting, grooming and feeding behaviors [55]. The bearing of the GPS antenna when facing 90° was chosen randomly. At all remaining sites (T1-T25, S1 Table) units were deployed for one day only (0°). Information stored for each successful GPS fix included fix number, fix date, fix time, latitude, longitude, number of satellites, and HDOP. We obtained the precipitation (mm) and mean temperature (°C) from Gvarv meteorological station (Meteorological Institute of Norway; URL: https://www.met.no/), located in the middle of our study area, for each test site on the day we conducted the test. Additionally, we used GPS data collected from 58 beavers (48 beavers were tagged with the G1G 134A, six with the TGB-317/315GX, and four with the PinPoint 75 micro) from 2009–2016 [48]. Beavers were trapped at night from a boat using landing nets, and GPS units were glued to the lower back using a two component resin [46], with the GPS antenna facing skyward when the beaver was swimming [55]. For details on capture, handling and GPS tagging see Graf, Mayer [47] and Steyaert, Zedrosser [46].

### Ethical statement

All trapping and handling procedures were approved by the Norwegian Experimental Animal Board (FOTS id 742, id 2170, 2579, 4384, 6282, 8687) and the Norwegian Directorate for Nature Management (2008/14367 ART-VI-ID, archive code 444.5, 446.15/3, 14415), which also granted permission to conduct fieldwork in the study area. Our study met the ASAB /ABS Guidelines for the treatment of animals in research and teaching ASAB/ABS.

### Data analysis

We calculated LE as the Euclidean distance in meters between the GPS-measured location and the true location. FSR was calculated for each GPS day by dividing the number of successfully obtained fixes by the maximum number of possible fixes.

Canopy closure was positively correlated with vegetation height, tree diameter and basal area (Spearman correlation coefficient r > 0.6 in all cases). Thus, we only included canopy closure in the analysis. Further, because HDOP and the number of satellites used for a fix were highly correlated (r = -0.69), we only included the HDOP in the analysis.

To analyze the LE (dependent variable, log-transformed to meet the assumption of normality), we used a mixed-effects linear regression with a Gaussian distribution. For this analysis, we included all test sites, but only the days when units were placed facing skyward (0°). We included the HDOP, GPS model, slope, canopy closure, and the interaction of canopy closure x slope as fixed effects and the GPS unit as random intercept to control for non-independence of the data. To investigate FSR (dependent variable; using all test sites, but only days with units facing skyward), we used a mixed-effects logistic regression with a binomial error distribution. We included the GPS model, mean daily temperature, daily precipitation (mm), canopy closure, slope, and the interaction of canopy closure x slope as fixed effects and the GPS unit as random intercept (we did not include more interactions to avoid overfitting the models). To investigate the effect of the GPS angle (simulating body posture) on LE and FSR, we analyzed the subset of data where we changed the angle of the GPS antenna (11 sites: T26-T36, S1 Table) using the same model structure, but including the GPS angle (0° versus 90°) and the interaction of GPS model x GPS angle as fixed effects. Finally, to study the influence of animal behavior, we assessed differences in FSR between stationary tests (201 GPS days) and GPS units deployed on 54 beavers (771 GPS days) using an unpaired t-test.

Model selection was based on Akaike’s Information Criterion for small sample sizes (AIC_c_ values, Table 1), selecting the model with the lowest AIC_c_ value [56]. We used the dredge function in R package MuMIn [57] to create a set of candidate models including all possible combinations of fixed effects and the above mentioned interactions. If ΔAIC_c_ was < 4 in two or more models, we performed model averaging [58]. Parameters that included zero within their 95% confidence intervals (CI) were considered as uninformative [59]. Data are shown as mean ± standard deviation (SD) unless otherwise stated. All statistical analyses were carried using the free software R 3.2.5 [60].

**Table 1:** The most parsimonious models within ΔAIC_c_ < 10 for the analysis of 1) location error, and 2) fix success rate for stationary tests of three GPS models. The GPS unit was included as random effect in all analyses. The analyses were based on all test sites, but excluding days when the GPS antenna was tilted by 90°.

Model	df	logLik	AIC_c_	delta AIC_c_	AIC_c_ weight
*1) Location error*					
GPS model + HDOP + Slope + Canopy closure + Slope x Canopy closure	9	-9787	19591	0.00	0.70
GPS model + HDOP + Slope + Canopy closure	8	-9789	19594	2.09	0.25
HDOP + Slope + Canopy closure + Slope x Canopy closure	7	-9791	19597	5.57	0.04
HDOP + Slope + Canopy closure	6	-9794	19599	7.69	0.02
*2) Fix success rate*					
GPS model + Precipitation + Slope + Temperature + Canopy closure	8	-708.4	1433.7	0.00	0.54
GPS model + Precipitation + Slope + Temperature + Canopy closure + Slope x Canopy closure	9	-707.8	1434.7	1.02	0.33
Precipitation + Slope + Temperature + Canopy closure	6	-712.5	1437.4	3.76	0.08
Precipitation + Slope + Temperature + Canopy closure + Slope x Canopy closure	7	-711.8	1438.4	4.71	0.05

## Results

### Location error

In total, we obtained 9,624 location fixes from 33 stationary test sites (we did not obtain any fixes underwater and underground). Within these fixes, 96.8% were 3-D locations (≥ 4 satellites) and 3.2% 2-D locations (3 satellites). The LE associated with 3-D fixes was significantly smaller compared to 2-D fixes (15.1 ± 19.8 m versus 33.7 ± 53.2 m, t-test: *t* = 4.47; p < 0.001). The mean number of obtained satellites per fix was 5.4 ± 1.5 (range: 3–12 satellites) and the mean HDOP was 2.6 ± 2.1 (range: 0.6–45.6).

**Fig 2:**
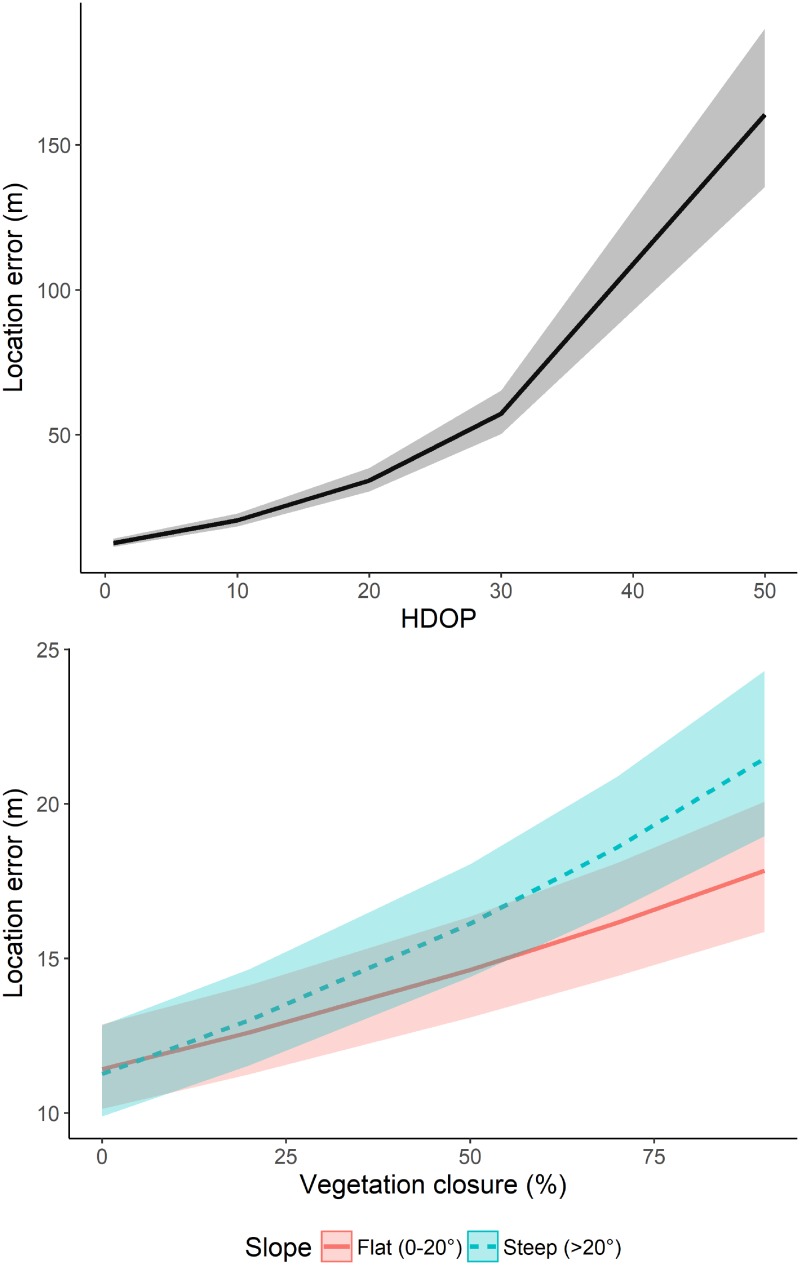
The effect of horizontal dilution of precision (HDOP, upper graph) and the interaction of canopy closure x slope (lower graph) on the location error of GPS receivers during stationary tests. Slope was categorized into ‘flat’ (0–20°) and ‘steep’ (> 20°) for reasons of better visualization.

**Fig 3:**
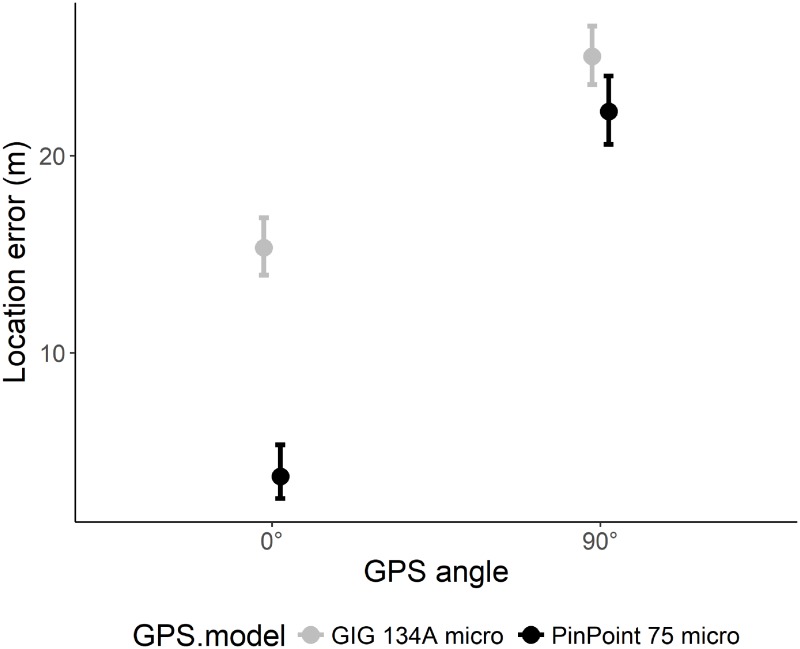
The effect of the GPS angle (0° versus 90°) on the location error of two models of GPS receivers during stationary tests.

The mean LE of all GPS positions was 15.7 ± 21.9 m, and ranged between 0.0 and 364.5 m (S1 Fig). When comparing between models (excluding days when the GPS antenna was tilted by 90°), the LE of the GIG 134A micro (14.8 ± 16.2 m) was significantly larger compared to the TGB-317/315GX (13.5 ± 20.1 m) and the PinPoint 75 micro (13.8 ± 20.1 m; ANOVA: F = 3.72, p = 0.024). The LE increased with increasing HDOP (Table 2, Fig 2). Further, the interaction of canopy closure x slope revealed that the LE increased with increasing canopy closure and more so in steeper slopes (Table 2, Fig 2). Analyzing the subset of sites where we shifted the GPS angle showed that the LE was markedly lower when the GPS receiver was facing skyward, i.e., at 0° compared to when tilted by 90° (14.2 ± 18.6 versus 22.5 ± 31.6 m; Estimate ± SD = 0.49 ± 0.04; 95% CI: 0.42; 0.57). The LE of GPS model PinPoint 75 micro increased more when tilted by 90° compared to the GIG 134A micro (Fig 3). Removing HDOP values > 5 and < 4 available satellites led to a data loss of 9% and improved the mean LE by 1.7 m to 14.0 ± 17.0 m. This procedure removed many outliers (mean ± SD of removed GPS positions: 69.0 ± 21.6 m), but not all. When removing HDOP values > 4 and < 5 available satellites, the LE further improved to 11.9 ± 13.1 m and resulted in a data loss of 27.9%.

**Table 2:** Effect size (β), standard error (SE), and lower (LCI) and upper (UCI) 95% confidence intervals of explanatory variables for the analysis of 1) location error, and 2) fix success rate for stationary tests of three GPS models. We performed model averaging of best models (ΔAIC_c_ < 4) to estimate the effect size of each variable. Informative parameters are presented in bold.

Variable	β	SE	LCI	UCI
*1) Location error*				
**GPS model (TGB-317/315GX)**	**-0.59**	**0.12**	**-0.84**	**-0.35**
GPS model (TGB PinPoint 75 micro)	-0.20	0.12	-0.45	0.04
**HDOP**	**0.16**	**0.00**	**0.16**	**0.17**
**Slope**	**0.10**	**0.01**	**0.08**	**0.12**
**Canopy closure**	**0.23**	**0.01**	**0.22**	**0.25**
**Slope x Canopy closure**	**-0.02**	**0.01**	**-0.04**	**-0.001**
*2) Fix success rate*				
**GPS model (TGB-317/315GX)**	**-1.83**	**0.67**	**-3.15**	**-0.52**
**GPS model (TGB PinPoint 75 micro)**	**-2.22**	**0.66**	**-3.52**	**-0.92**
**Precipitation**	**0.34**	**0.08**	**0.19**	**0.49**
**Slope**	**-0.20**	**0.06**	**-0.31**	**-0.09**
**Temperature**	**0.77**	**0.09**	**0.60**	**0.95**
**Canopy closure**	**0.32**	**0.07**	**0.18**	**0.46**
Slope x Canopy closure	-0.08	0.07	-0.21	0.06

### Fix success rate

**Fig 4:**
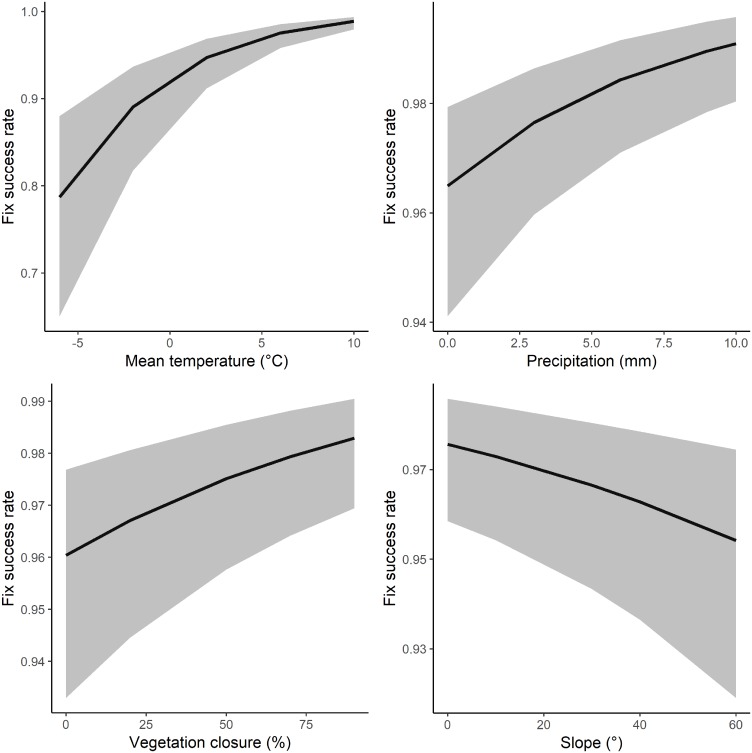
The effect of mean temperature (top left), precipitation (top right), canopy closure (bottom left) and slope (bottom right) on the fix success rate of GPS receivers during stationary tests.

**Fig 5:**
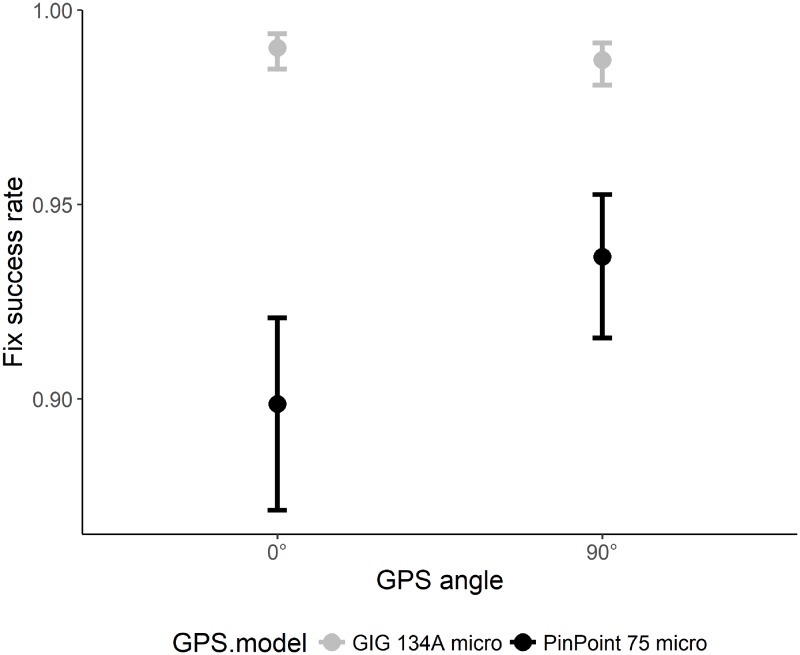
The effect of the GPS angle (as measure of body posture) on the fix success rate of two GPS receiver models during stationary tests.

The overall FSR of all units during stationary tests was 94.2 ± 14.8%. FSR differed between models with the GIG 134A micro (98.1 ± 1.8) performing better compared to the PinPoint 75 micro (90.5 ± 20.4) and TGB-317/315GX (93.7 ± 15.1), respectively (Table 2). FSR increased with increasing temperature, precipitation, and canopy closure, and decreased with increasing slope (Table 2, Fig 4). The interaction of canopy closure x slope was uninformative. When analyzing the data for the model GIG 134A micro only, only slope had a negative effect on FSR and all other variables were uninformative (results not shown). A separate analysis of the 11 sites where we shifted the GPS angle revealed that the FSR of the model GIG 134A micro was not affected by the GPS angle, but the PinPoint 75 micro had an increased FSR when the units were tilted by 90° (Fig 5). Moreover, none of the GPSs recorded positions underwater or underground.

The FSR of GPS units that were deployed on beavers was significantly lower compared to the stationary tests (86.2 ± 10.3% versus 94.2 ± 14.8%; t-test: *t* = -17.11; p < 0.001). The FSR of GPS units deployed on beavers was significantly different between two GPS receiver models, with the GIG 134A micro performing better compared to the TGB-317/315GX (86.6% versus 83.5%, t-test: t = 2.13; p = 0.035). We did not obtain any field data from the PinPoint 75 micro as we only recovered one unit, which did not record any data (we did not find the other three units).

## Discussion

When studying animal movement and habitat use, it is crucial to know the quality of obtained GPS data and to understand the error-inducing mechanisms [27]. As predicted, LE depended on both habitat and technical variables as well as body posture, which might lead to an erroneous categorization of GPS positions when studying habitat use. Data screening can help to remove GPS positions with large LE [26], and alternative methods, like the combination with accelerometers, can be used to avoid wrong habitat assignment. The mean FSR for all GPS models during stationary tests was > 90%, which was higher compared to the FSR of GPS units deployed on beavers. This difference was likely caused by animal behavior. We discuss the possibility of using FSR to calculate activity times of burrow-living animals.

### Location error

The mean LE was 15.7 m, in accordance with the typical range (10 to 30 m) reported by other studies [26, 27, 61]. The results from our stationary tests indicate that LE is affected by both technical (HDOP and GPS model) and habitat (canopy closure and slope) variables, as well as animal body posture (GPS angle). LE increased with increasing HDOP, and removing 2-D positions and HDOP values > 5 reduced the LE, removing many, but not all outliers. Removing lower HDOP values and positions < 5 satellites further increased the LE, but led to a large data loss. Hence, we suggest to follow the guideline by Lewis, Rachlow [26], i.e. removing 2-D positions and DOP values > 5. Further, LE differed among the three GPS models. Nevertheless, this difference was within one meter, making the LE of the three models comparable. When simulating varying body posture by changing the GPS angle, we found that the LE was markedly increased when the GPS antenna was tilted by 90°, consistent with previous studies testing collar position and orientation [16, 62]. LE also increased in locations with dense canopy cover and more so in steeper slopes. These results suggest that there will be a larger LE associated with land positions compared to GPS positions on water, because the GPS is directly facing skyward when an animal is swimming (depending on the attachment method) and because there is no slope and less obstruction by vegetation. Canopy closure varies with the vegetative season, potentially also leading to variation of the LE associated with land positions over the course of the year. Beavers are often sitting when on land [55], and they spend much time foraging in forest [46], i.e., in areas with dense vegetation cover, suggesting that the effects of GPS angle, slope and canopy closure amplify each other. Further, we previously found that older beavers spend comparatively more time on land [47], which indicates that individual differences in animal behavior might also affect LE (in this case via an altered body posture and increased canopy closure on land). Generally, beavers in our study area (equipped with the GPS model GIG 134A micro) were shown to spend most of the time close to shore (on average 15 m), both when being on land and in water [47]. This distance is similar to the LE obtained in our study, and could result in inaccurate estimates when studying habitat use, resource selection [1, 27], and when calculating behavioral time budgets due to misclassification of land and water positions, respectively. This is important, because beavers (and other semi-aquatic animals) are in water or on land for very different purposes. For example, beavers use water to travel [48, 63] and spend much of their time on land foraging [52]. Another source of uncertainty are map errors that in the case of riparian habitats can vary depending on the water level.

### Fix success rate

The FSR in our study was generally high (> 90%) and varied among models and with canopy closure, slope and weather conditions. The FSR of the GPS model GIG 134A micro was close to 100% and was more robust compared to the other two models; e.g., it was not affected by a varying GPS angle or weather conditions. Surprisingly, canopy closure and precipitation had a positive effect on the FSR of the models TGB-317/315GX and PinPoint 75 micro, a finding previously reported for canopy closure [32]. Although canopy closure, slope and precipitation had an effect on the FSR, their effect was comparatively small as FSR remained > 93% in all cases. In contrast, temperatures < 0 °C led to a marked decrease in FSR for the models TGB-317/315GX and PinPoint 75 micro, suggesting that FSR decreases during the colder months of the year.

The FSR of GPS units deployed on beavers was 86%, lower than in stationary tests, a finding similar to other studies [14, 20, 64]. Nevertheless, the FSR was higher or comparable to results reported for GPS devices deployed on other mammals. For example, a FSR of 85% was recorded for white-tailed deer (*Odocoileus virginiaunus*) [28], 81% for pygmy rabbits (*Brachylagus idahoensis*) [65], and 68% for otters [32]. The reduction of performance between stationary tests and field trials might be explained by animal behavior [22, 66]. GPS units did not record any positions underground as reported previously [65, 67]. Thus, the most likely explanation for the reduced FSR was, because beavers return to their lodge/den during their activity period. Sharpe and Rosell [52] reported that beavers spend ca. 32% of their time budget inside the lodge, and based on acceleration data we estimated that beavers spend ca. 10% of their active time inside the lodge (MM, unpublished results). The large proportion of time spent inside the lodge found by Sharpe and Rosell [52] might be owed to an observer bias, because these beavers were observed throughout the night from a motorboat using spotlights, which might have impacted the beavers’ natural behavior. This is a good example how GPS technology can be used to reduce observer bias, and to estimate principal activity periods of animals that use burrows or dens as shelter. Further, GPSs did not record any positions underwater (independent of submersion depth). However, beavers typically dive for short periods of time (on average < 30 sec) and spend < 3% of their nightly activity on diving [68]. GPS receivers attempted to acquire locations for 3 min (the PinPoint 75 micro for 70 sec). Therefore, diving activity probably had little influence on the FSR. For other semi-aquatic species that spend more time underwater, e.g. crocodiles [69] and turtles [70], the FSR could potentially be used to quantify the time spend in/under water after initial calibration and depending on the fix rate.

### Conclusions

The LE in our study was on average 15.7 m, which is similar to the average distance that beavers stay from the shoreline [47], making it hard to reliably categorize GPS positions into being in water versus on land. Data screening can improve the LE via removing 2-D positions and positions with HDOP values > 5 [26]. Additionally, the use of buffer areas along the shoreline could be used to remove uncertain GPS positions. For example, a buffer zone the size of the LE (i.e., 15 m in our case) could be created to remove all GPS positions therein. In the case of the beaver, this is not feasible, because it would result in the removal of 60% of all data (results not shown). However, it might be possible for species that forage further from the water, e.g. hippopotami (*Hippopotamus amphibius*) [71].

We suggest that the best solution to identify land versus water positions is using the GPS unit in combination with an accelerometer. Graf, Wilson [55] used accelerometers attached on the lower back of beavers (same location as GPS units in this study) to record changes in body posture and body movement, which were then used to identify different behaviors. This enables to link GPS positions to specific behavior, e.g. if an animal was sitting, walking or swimming. Consequently, this could be used to assign GPS positions to land or water, respectively, allowing for more precise estimates of habitat use and behavioral time budgets, and could potentially also resolve the problem of inaccurate maps and changing water levels. Further, as GPS devices in our study were placed at the same body position as the accelerometers, we could relate specific behaviors to an increased LE, which would allow controlling for inaccurate positions. For example, when beavers are standing the GPS is turned 90° [55], leading to an increased LE. To increase battery life, GPS units could be programmed to only take positions when an animal is active, determined by acceleration data and accelerometers could be programmed to only record data at the same time as the GPS obtains a position. We conclude that the use of GPS telemetry is an effective tool to collect detailed location data suitable to study animal home range size, spatial movement patterns, space use, and habitat selection in semi-aquatic animals, although some limitations still exist. Future research should aim to quantify how to increase the certainty of location data by combining GPS units with other data loggers.

## Supporting information

S1 Table Overview of the 36 test sites in Southeast Norway showing the fix success rate and location error separately for the three GPS models. Model TGB-317/315GX was only deployed at test sites T1-T25. NA values indicate technical problems (no data obtained).
(DOCX)

S1 Fig Frequency histogram of the GPS location error (m) obtained during 33 stationary tests. (DOCX)
