# Supplementary material for: Performance of GPS units for deployment on semiaquatic animals
Source: PLoS One. 2018 Dec 6;13(12):e0207938. doi: 10.1371/journal.pone.0207938 (PMC6283466; doi:10.1371/journal.pone.0207938)
Supplement: S1 Table — Model TGB-317/315GX was only deployed at test sites T1-T25. NA values indicate technical problems (no data obtained). (DOCX) [file pone.0207938.s001.docx]

**Supporting information**

**S1 Table.** Overview of the 36 test sites in Southeast Norway showing the fix success rate and location error separately for the three GPS models. Model TGB-317/315GX was only deployed at test sites T1-T25. NA values indicate technical problems (no data obtained).

|  |  |  |  | Fix success rate | | | Location error (m) | | |
| --- | --- | --- | --- | --- | --- | --- | --- | --- | --- |
| Test site | Slope | Canopy closure | Comment | GIG 134A micro | PinPoint 75 micro | TGB-317/315GX | GIG 134A micro | PinPoint 75 micro | TGB-317/315GX |
| T1 | 2 | 0 |  | 1.00 | 1.00 | 0.60 | 10.3 ± 6.5 | 7.5 ± 11 | 8.8 ± 8.6 |
| T2 | 3 | 0.13 |  | 0.96 | 1.00 | 0.67 | 9.4 ± 7.8 | 4.4 ± 2.9 | 8.1 ± 8.6 |
| T3 | 3 | 0.94 |  | 0.99 | 0.98 | 0.99 | 21.5 ± 16.2 | 20.2 ± 23.5 | 24.4 ± 22.4 |
| T4 | 3 | 0.7 |  | 0.98 | 0.99 | 0.99 | 15.4 ± 11.5 | 14.9 ± 18.3 | 15.2 ± 13.6 |
| T5 | 28 | 0.05 |  | 0.99 | 1.00 | 0.98 | 10.4 ± 8.9 | 8.6 ± 9.3 | 5.5 ± 5.7 |
| T6 | 30 | 0.31 |  | 0.98 | 1.00 | 0.99 | 11.6 ± 8 | 10.2 ± 10.4 | 11.4 ± 16.5 |
| T7 | 34 | 0.83 |  | 0.98 | 0.98 | 0.79 | 17.9 ± 13.9 | 18.9 ± 20.8 | 19.8 ± 18.8 |
| T8 | 1 | 0.7 |  | 0.99 | 0.99 | 0.82 | 12.7 ± 10.3 | 16.4 ± 16.3 | 19.1 ± 19.7 |
| T9 | 44 | 0.83 |  | 0.96 | 1.00 | 0.93 | 23.1 ± 17.1 | 19.8 ± 20.1 | 30.7 ± 31.1 |
| T10 | 39 | 0.57 |  | 0.95 | 0.99 | 0.98 | 24.6 ± 20.1 | 17 ± 20.1 | 14.7 ± 12.1 |
| T11 | 39 | 0.38 |  | 0.98 | 1.00 | 0.95 | 18.7 ± 15.4 | 16.9 ± 18.5 | 15.8 ± 17.9 |
| T12 | 5 | 0.54 |  | 1.00 | 1.00 | 0.99 | 16.9 ± 13.7 | 20.2 ± 20 | 18.4 ± 20.5 |
| T13 | 7 | 0.08 |  | 0.98 | 0.50 | 1.00 | 15.2 ± 12.8 | 9.1 ± 7.5 | 9.5 ± 9.6 |
| T14 | 2 | 0.69 |  | 0.97 | 1.00 | 1.00 | 17.2 ± 16.1 | 16.3 ± 40.9 | 13.8 ± 27.7 |
| T15 | 2 | 0.84 |  | 0.99 | 1.00 | 0.98 | 14.8 ± 13.2 | 16.6 ± 25.4 | 12.9 ± 16.8 |
| T16 | 10 | 0.08 |  | 1.00 | 0.74 | 1.00 | 13 ± 12.4 | 9.4 ± 10.5 | 4.8 ± 3.8 |
| T17 | 6 | 0.14 |  | 0.99 | 0.34 | 1.00 | 8.1 ± 6.7 | 11.6 ± 13 | 8.1 ± 25.4 |
| T18 | 20 | 0.64 |  | 0.99 | 0.99 | 0.98 | 17.6 ± 20.9 | 13.9 ± 13.3 | 14.4 ± 17.7 |
| T19 | 13 | 0.91 |  | 0.98 | 1.00 | 0.99 | 17.7 ± 15 | 20.2 ± 19.7 | 17.3 ± 27 |
| T20 | 20 | 0.06 |  | 0.97 | 1.00 | 1.00 | 11.3 ± 7.2 | 7.5 ± 6.2 | 8.6 ± 24.3 |
| T21 | 0 | 0 |  | 1.00 | 1.00 | 1.00 | 12 ± 9 | 8.2 ± 11.4 | 13 ± 25.6 |
| T22 | 0 | 0 |  | 0.99 | 1.00 | 0.99 | 6.7 ± 6 | 4.8 ± 3.4 | 3.2 ± 5.2 |
| T23 |  |  | underwater | 0.00 | 0.00 | 0.00 | - | - | - |
| T24 |  |  | underwater | 0.00 | 0.00 | 0.00 | - | - | - |
| T25 |  |  | underground | 0.00 | 0.00 | 0.00 | - | - | - |
| T26 | 0 | 0.71 |  | 0.98 | NA |  | 8.2 ± 6 | NA |  |
| T27 | 28 | 0.75 |  | 0.97 | 0.73 |  | 11 ± 15.3 | 8.7 ± 8.9 |  |
| T28 | 54 | 0.77 |  | 0.98 | 0.30 |  | 15.5 ± 15.2 | 15.6 ± 15 |  |
| T29 | 0 | 0.1 |  | 0.98 | NA |  | 10.5 ± 8.7 | NA |  |
| T30 | 41 | 0.95 |  | 0.98 | 1.00 |  | 17.2 ± 19.9 | 14.4 ± 15 |  |
| T31 | 30 | 0.8 |  | 0.99 | NA |  | 12.3 ± 14 | 11.2 ± 7.6 |  |
| T32 | 56 | 0.92 |  | 0.97 | 0.61 |  | 17.8 ± 21.4 | 13.8 ± 13.3 |  |
| T33 | 0 | 0.08 |  | 0.98 | 1.00 |  | 13.7 ± 10.5 | 8.9 ± 12.3 |  |
| T34 | 15 | 0.49 |  | 0.96 | 1.00 |  | 26.1 ± 46.2 | 25.1 ± 48.1 |  |
| T35 | 6 | 0.49 |  | 0.98 | 1.00 |  | 21.4 ± 21.4 | 16.8 ± 15.5 |  |
| T36 | 8 | 0.05 |  | 1.00 |  |  | 11 ± 7.5 | 11.8 ± 18.7 |  |
