# Supplementary material for: Performance of GPS units for deployment on semiaquatic animals
Source: PLoS One. 2018 Dec 6;13(12):e0207938. doi: 10.1371/journal.pone.0207938 (PMC6283466; doi:10.1371/journal.pone.0207938)
Supplement: S1 Fig — (DOCX) [file pone.0207938.s002.docx]

**Supporting information**


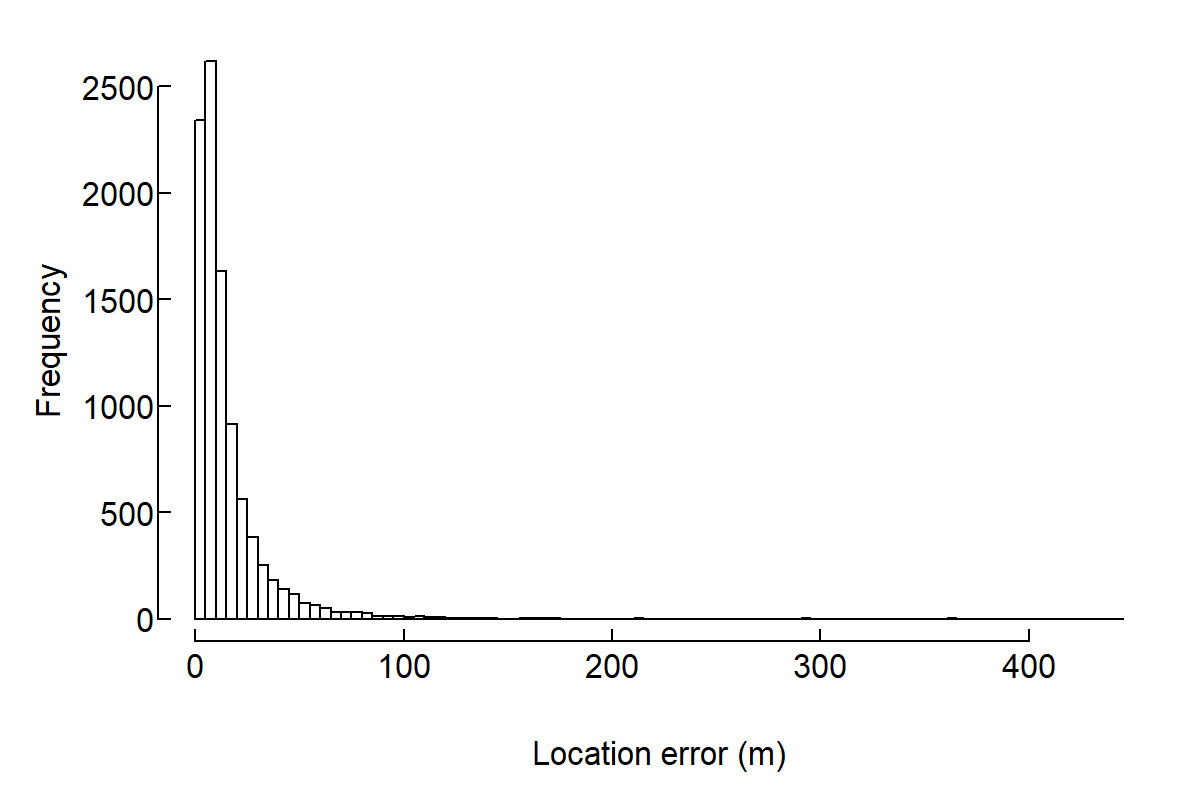


**S1 Fig.** Frequency histogram of the GPS location error (m) obtained during 33 stationary tests.
